# Improving Screening Uptake among Breast Cancer Survivors and Their First-Degree Relatives at Elevated Risk to Breast Cancer: Results and Implications of a Randomized Study in the State of Georgia

**DOI:** 10.3390/ijerph17030977

**Published:** 2020-02-04

**Authors:** Joseph Lipscomb, Cam Escoffery, Theresa W. Gillespie, S. Jane Henley, Robert A. Smith, Toni Chociemski, Lyn Almon, Renjian Jiang, Xi Sheng, Michael Goodman, Kevin C. Ward

**Affiliations:** 1 Rollins School of Public Health, Emory University; Atlanta, GA 30322, USA; cescoff@emory.edu (C.E.); Robert.smith@cancer.org (R.A.S.); tchocie@emory.edu (T.C.); malmon@emory.edu (L.A.); Renjian.jiang@emory.edu (R.J.); xi_sheng92@163.com (X.S.); Mgoodm2@emory.edu (M.G.); kward@emory.edu (K.C.W.); 2 Winship Cancer Institute of Emory University; Atlanta, GA 30322, USA; tgilles@emory.edu; 3 Department of Surgery and Department of Hematology and Medical Oncology, Emory University School of Medicine; Atlanta, GA 30322, USA; 4 Division of Cancer Prevention and Control, U.S. Centers for Disease Control and Prevention; Atlanta, GA 30341, USA; Skh3@cdc.gov; 5 Cancer Control Program, American Cancer Society; Atlanta, GA 30303, USA

**Keywords:** community-based research, population-based genetic risk screening, breast cancer survivors, first-degree relatives, disease screening, guideline adherence, cancer registries

## Abstract

Women diagnosed with breast cancer at a relatively early age (≤45 years) or with bilateral disease at any age are at elevated risk for additional breast cancer, as are their female first-degree relatives (FDRs). We report on a randomized trial to increase adherence to mammography screening guidelines among survivors and FDRs. From the Georgia Cancer Registry, breast cancer survivors diagnosed during 2000–2009 at six Georgia cancer centers underwent phone interviews about their breast cancer screening behaviors and their FDRs. Nonadherent survivors and FDRs meeting all inclusion criteria were randomized to high-intensity (evidence-based brochure, phone counseling, mailed reminders, and communications with primary care providers) or low-intensity interventions (brochure only). Three and 12-month follow-up questionnaires were completed. Data analyses used standard statistical approaches. Among 1055 survivors and 287 FDRs who were located, contacted, and agreed to participate, 59.5% and 62.7%, respectively, reported breast cancer screening in the past 12 months and were thus ineligible. For survivors enrolled at baseline (*N* = 95), the proportion reporting adherence to guideline screening by 12 months post-enrollment was similar in the high and low-intensity arms (66.7% vs. 79.2%, *p* = 0.31). Among FDRs enrolled at baseline (*N* = 83), screening was significantly higher in the high-intensity arm at 12 months (60.9% vs. 32.4%, *p* = 0.03). Overall, about 72% of study-eligible survivors (all of whom were screening nonadherent at baseline) reported screening within 12 months of study enrollment. For enrolled FDRs receiving the high-intensity intervention, over 60% reported guideline screening by 12 months. A major conclusion is that using high-quality central cancer registries to identify high-risk breast cancer survivors and then working closely with these survivors to identify their FDRs represents a feasible and effective strategy to promote guideline cancer screening.

## 1. Introduction

An ongoing challenge in breast cancer screening is improving adherence to recommendations for regular testing, especially among those at elevated risk of disease. Of note, women diagnosed with breast cancer at a relatively young age (≤45 years) have about a 4-fold risk of a recurrent or second primary breast cancer [1] and a 1.5 to 2-fold risk of any subsequent cancer, compared with the general at-risk population [2,3,4]. Women diagnosed with bilateral breast cancer (synchronous or metachronous) at any age have a 1.2–2.0 relative risk of recurrence [5]. Both genetic factors (e.g.; BRCA1 or BRCA2 mutations) and environmental factors (e.g.; radiation exposure to breast/chest in childhood or adolescence) are thought to be associated with an early-age diagnosis [6] and possibly also bilateral disease, but these matters of etiology remain active areas for further investigation.

Since women who are diagnosed at a young age or with bilateral breast cancer are at high risk of being diagnosed with breast cancer again, they should receive surveillance mammography on an annual basis at least through age 75 years [1]. Moreover, female first-degree relatives (FDR), including mother, sisters, and daughters, of these high-risk breast cancer survivors are also at high risk of developing breast cancer, with relative risks ranging from 2.1−4.0, depending on other risk factors [1,7]. These close relatives should all receive age-appropriate breast cancer screening [1]. It thus can be reasonably inferred that an early age of diagnosis is a significant biologic indicator of an elevated risk to subsequent breast cancer, both for the survivor and her FDR(s).

From a population health perspective, one potentially feasible and effective approach for identifying such breast cancer survivors is to capitalize on information available from population-based cancer registries. In the U.S.; the National Program of Cancer Registries (NPCR) within the Centers for Disease Control and Prevention (CDC) currently provides financial and scientific support for such registries within 46 states, the District of Columbia, Puerto Rico, and additional U.S. territories. At the same time, there is no registry or other systematic process now available for identifying close relatives of cancer survivors. Consequently, the cancer survivor herself is the pivotal information source, so that any efforts to reach out to close relatives in a targeted way must engage the cancer survivor [8,9,10].

Following release of the Landmark Institute of Medicine report, *From Cancer Patient to Cancer Survivor: Lost in Transition* [11], there is now a broad consensus in the U.S. cancer community that every patient completing cancer-directed therapy should receive a survivorship care plan that provides recommendations on surveillance for future cancers, as well as guidance on health behaviors to enhance quality of life and longevity [12,13,14,15]. That said, the optimal approach for reaching out to cancer survivors who did not receive a survivorship care plan upon completion of initial cancer therapy or who received such a plan but without any intervention to promote adherence, remains unclear. Additionally, to our knowledge, none of the prominent survivorship care plan templates [16,17,18] provide a direct pathway for informing cancer survivors that their close relatives may be at elevated cancer risk and encouraging them to discuss cancer screening with their primary care providers.

We report here the findings from a CDC-supported study based in the state of Georgia to test the feasibility and effectiveness of strategies for using a population-based cancer registry to launch educational interventions to increase breast cancer screening among breast cancer survivors at elevated risk for additional breast cancer, as well as breast cancer screening among their FDRs. To our knowledge, ours is the first population-based study to investigate whether interventions can increase breast cancer screening among breast cancer survivors and their own FDRs.

## 2. Materials and Methods

### 2.1. Study Participants

**Figure 1:**
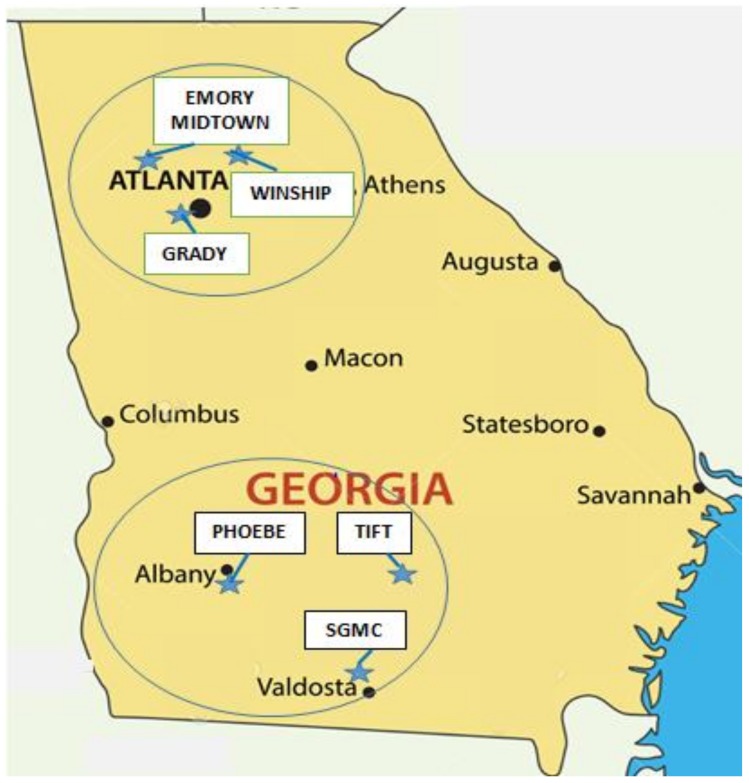
Cancer Centers in metropolitan Atlanta and Southwest Georgia assisting in study recruitment of breast cancer survivors. SGMC = South Georgia Medical Center.

To identify and enroll breast cancer survivors and their FDRs, the study team partnered in 2011 with the Georgia Cancer Registry based in the Georgia Department of Public Health; the Georgia Center for Cancer Statistics at Emory’s Rollins School of Public Health; the Cancer Coalition of South Georgia based in Albany; the CDC’s Division of Cancer Prevention and Control; 3 cancer centers in the metropolitan Atlanta area (Winship Cancer Institute at Emory, Emory Midtown Cancer Center, and the Georgia Cancer Center for Excellence at Grady Memorial Hospital); and 3 cancer centers in the southwest region of Georgia (Phoebe Putney Cancer Center in Albany, Tift Regional Medical Center in Tifton, and South Georgia Medical Center in Valdosta). All 6 cancer centers are Commission-on-Cancer (CoC)-approved, and their locations in Georgia are displayed in Figure 1.

Recruitment of Breast Cancer Survivors: We used the Georgia Cancer Registry to identify all women diagnosed from 2002 through 2009 with either a first primary breast cancer at age 45 years or younger or with bilateral breast cancer at any age who received all or part of their first course of therapy at one of the 6 participating cancer centers and were alive in 2012 based on follow-up data from the Georgia Cancer Registry. The focus was on women treated with curative intent, so we included those diagnosed with a Surveillance, Epidemiology, and End-Results (SEER) Summary Stage of in situ, localized, or regional breast cancer (and thus excluded those with distant metastases or unknown stages). To determine the most current residential address and telephone number for each survivor, we combined information from the Georgia Cancer Registry, the cancer center where she was treated, and electronic search engines (e.g.; Accurint) used by the Georgia Center for Cancer Statistics in its role as manager of data operations for the state registry.

Each breast cancer survivor with adequate contact information received a mailed letter of invitation to participate in the study from her treating cancer center on official letterhead and signed by the center’s leadership. The survivor was asked to respond by phone to the study coordinator with an expression of interest; if no response was received within 2 weeks of the mailing, we proceeded with telephone follow-up (to a maximum of 10 calls over a 1-month period). Each survivor successfully reached was provided a brief, verbal summary of the study; if she wished to participate, her inclusion criteria were evaluated (see next paragraph) and, if met, she was asked to give informed consent via the phone call, with a copy of the consent form subsequently mailed to her.

In addition to being diagnosed at age 45 years or younger or having a bilateral disease, inclusion criteria included being fluent in English, having no comorbid conditions that would prevent completion of the study, and not having received a mammogram within the past 12 months. Women reporting other radiographic tests for breast cancer (magnetic resonance imaging (MRI), positron emission tomography (PET), or computed tomography (CT)) over the past 12 months were also excluded, since these tests―while not included in current screening recommendations for survivors with adequate breast cancer tissue―do demonstrate the survivor’s commitment to breast cancer screening. We sought to exclude any survivor with a confirmed bilateral mastectomy because our educational interventions (see *Interventions*) were geared toward mammography, which is not the appropriate surveillance pathway for women with both breasts removed. We also excluded women with bilateral breast cancer who were, at the time of the interview, older than age 75 years. Hence, our aim was to identify high-risk breast cancer survivors who could benefit from adherence to surveillance mammography.

For each survivor thus engaged by phone (whether or not ultimately enrolled in the study), we briefly stated that her female FDRs “may be at increased risk” for breast cancer, and we asked her to provide contact information for each of her female FDRs so that we could invite them to join the study. The process of obtaining FDR contact information from these breast cancer survivors ended up proceeding in two phases. Initially, we sought FDR information only from survivors who met inclusion criteria and were successfully enrolled. However, to boost FDR enrollment, we subsequently attempted to recontact all study-ineligible survivors to obtain contact information for their FDRs.

Recruitment of First-Degree Relatives: Each identified FDR received a mailed letter of invitation to participate in the study (signed by the principal investigator on Emory University letterhead). Similar to the procedure for survivors, if the FDR did not respond to the study coordinator within 2 weeks of the mailing, we initiated telephone follow-up (maximum of 10 calls over a 1-month period). Each FDR successfully reached by phone was provided a brief, verbal summary of the study; if she wished to participate, her inclusion criteria were evaluated, and, if eligible, she was asked to provide informed consent, with a copy of the completed consent form then mailed to her.

Inclusion criteria for FDRs were: being the survivor’s mother, sister (full or half-biological), or daughter; age 18–75 years; fluent in English; no comorbid conditions that would prevent completion of the study; not previously diagnosed with cancer; and had not received a mammogram (or other age-appropriate breast cancer screening) within the past 12 months.

In general, appropriate screening for the FDR was defined in terms of her own age, both in an absolute sense and in relation to the age when her breast cancer survivor relative was diagnosed. Specifically, an FDR was regarded as currently adherent to screening recommendations, and hence, ineligible, if (a) she was age 40–75 years or the difference between her current age and the age at which the breast cancer survivor had been diagnosed was 10 years or less and she had received a screening mammogram in the past 12 months or (b) she was age 18–40 years and more than 10 years younger than the age at which the breast cancer survivor had been diagnosed and, in the past 12 months, had received any breast screening exam, including a clinical breast exam, or reported being under surveillance by her primary care provider.

This algorithm was informed by recommendations from the National Comprehensive Cancer Network (NCCN) for screening women at “increased risk” of breast cancer [19] and tailored to meet the specific requirements of this study, with particular attention to the age of the FDR relative to the age of the survivor at her breast cancer diagnosis. To illustrate, if the survivor was age 43 at breast cancer diagnosis and the FDR was age 37 at interview, the FDR would be regarded as screening adherent if and only if she had received a mammogram in the past 12 months. If the FDR, instead, had been age 30 at interview, she would be regarded as screening adherent if she had received a clinical breast exam or otherwise reported being under surveillance by her primary care physician within the past 12 months.

Baseline Survey: Within two weeks of admission to the study (on average), each survivor and FDR completed a baseline survey by phone to confirm eligibility, collect sociodemographic information, and attempt to record the name and location of the woman’s primary care provider (PCP). Sociodemographic information included age, race/ethnicity, health insurance status, number of individuals in the household, marital status, highest grade of school completed, employment status, annual family income from all sources, and verification of mailing address. Each survivor and FDR successfully completing the baseline survey was regarded as officially enrolled in the study as of that date.

### 2.2. Interventions

Upon completion of the baseline survey, each survivor was randomized to either a low-intensity or high-intensity intervention. Each FDR whose survivor-relative was participating in the study was subsequently assigned to the same intervention as that survivor-relative to ensure that any communications between them would be informed by the same level and intensity of information about breast cancer screening. FDRs whose survivor-relative were not included in the study were simply randomized to an intervention arm.

Low-intensity: Within a week of randomization, survivors and FDRs assigned to the low-intensity arm were mailed the compact educational brochure, “Breast Cancer and You: What You Need to Know”, created by the CDC’s Division of Cancer Prevention and Control [20].

High-intensity: Within 4 weeks of randomization, the following multicomponent intervention was put in place for survivors and FDRs:

- The educational brochure was sent to each study participant by mail, typically within a week of randomization.
- A half-hour tailored telephone counseling session was conducted by a member of the study outreach team, in which the importance of breast cancer screening was emphasized, the woman’s “readiness for change” to engage in appropriate screening was evaluated [21], financial and physical barriers to screening were assessed, and recommendations for positive actions were developed and communicated. Additionally, each breast cancer survivor was urged to encourage her female FDRs to “talk with your doctor about… having regular breast screening tests…”. A written version of the telephone counseling script was subsequently mailed to the survivor, along with a separate note encouraging breast cancer screening.
- For study participants able and willing to identify a primary care provider, that PCP was mailed a packet containing the following: (1) a cover letter describing the study and notifying the PCP that a named study participant (who currently was not adherent to breast cancer screening recommendations) was a member of his/her practice; (2) a letter of support―signed by the presidents of the Georgia Chapter of the American College of Physicians, the Georgia Academy of Family Physicians, and the Georgia Chapter of the American College of Obstetrics and Gynecology, as well as by the medical director of the Cancer Coalition of South Georgia―that encouraged the PCP to note their patient’s elevated risk status in her medical record and engage in appropriate screening and follow-up; and (3) a “pink reminder form” with the study participant’s name and screening status for inclusion in her medical record.

Follow-up Surveys: To assess the impact of the interventions, we sought to conduct 3-month and 12-month follow-up surveys of each breast cancer survivor and FDR. Both surveys were conducted by phone by members of the study outreach team. For each follow-up survey, there was a target date for its administration in relationship to the participant’s date of study enrollment. Thus, for the 3-month survey, the target date for administration was 3 months from the date of study enrollment; likewise, for the 12-month survey. Typically, a team member would begin contacting a study participant two weeks prior to the survey target date and would continue pursuing the subject for a minimum of two weeks post the survey target date.

The 3-month survey asked the participant whether she had undergone mammography screening “within the last 3 months” and, similarly, the 12-month survey asked about mammography screening “within the past 12 months”. These concretely defined time intervals closely approximated the average time from study enrollment to survey interview in each case, respectively. Additionally asked was whether she had received a reminder or other communication from a health care provider about breast cancer screening since the study began; this allowed for the possibility that those in either arm of the trial had received encouragement from their PCP.

All telephone interviews with survivors and FDRs were conducted by the study outreach team comprising graduate research assistants at the Rollins School of Public Health who were all recruited, trained, and monitored by the study coordinator (Ms. Chociemski).

### 2.3. Statistical Analyses

All study data were entered into a project-specific REDCap database, and statistical analyses were conducted using SAS 9.4. For purposes of analysis, we regarded breast cancer survivors and FDRs as distinct groups, with data from each group analyzed separately rather than being pooled.

Thus, for breast cancer survivors and FDRs, in turn, we examined the impact of the intervention (high-intensity vs. low-intensity) on receipt of recommended breast cancer screening (adherent vs. not adherent) by the time of their 3-month follow-up, and then, by the time of their 12-month follow-up. Conclusions about the differential effectiveness of the interventions at each timepoint were based on a two-tailed chi-square test of difference in the proportions being screened, with *p* < 0.05 (type I error) regarded as significant.

Since the high-intensity intervention included outreach to the study participants’ primary care physicians, we also examined whether there was a significant between-intervention difference (*p* < 0.05) at 3 months and at 12 months in respondents reporting they had received a “reminder from your physician about getting a breast screening exam”.

Although participants were randomized, there remained the possibility that one or more covariates had values that were significantly imbalanced between arms. We tested for such imbalances at baseline, at 3 months, and at 12 months, for both survivors and FDRs. For any unbalanced covariate(s), we estimated a binary logistic regression model to examine the joint impact of the intervention (high-intensity vs. low-intensity) and the covariate(s) on the likelihood of screening adherence. The aim was to see whether the trial-based estimate of the intervention effect was significantly altered after accounting for the influence of any covariate imbalances [22].

As noted, all subjects gave informed consent for inclusion before they participated in the study. The study was conducted in accordance with the Declaration of Helsinki, and all protocols were approved by the Emory University Institutional Review Board (study #IRB00054239), the Georgia Department of Public Health IRB (project #140708), and the IRBs and research committees at the cancer centers (all of which used the Emory and GA DPH approvals as a basis for their approval decisions).

## 3. Results

### 3.1. Study Enrollment and Retention

**Figure 2:**
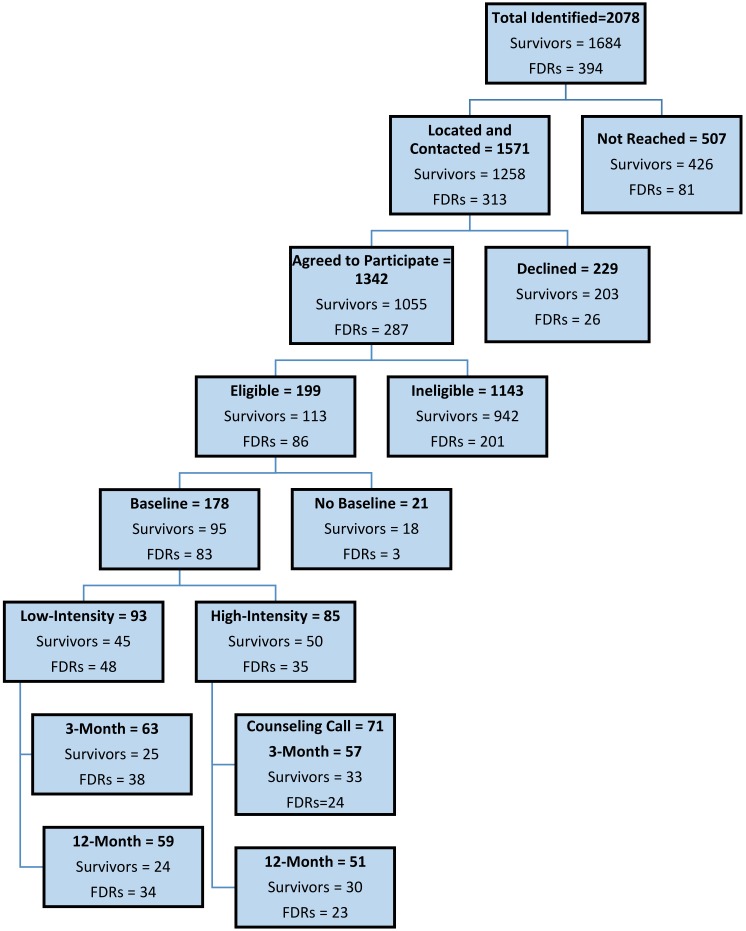
Recruitment and baseline enrollment of breast cancer survivors and their first-degree relatives (FDR) in metropolitan Atlanta and Southwest Georgia and retention for screening assessment purposes at 3 months and 12 months by intervention arms.

Recruitment of breast cancer survivors diagnosed during 2002–2009 began in May 2012, and telephone-based communications with survivors and FDRs continued through September 2014, as summarized in Figure 2 (CONSORT schema).

Breast Cancer Survivors: Of 1684 breast cancer survivors identified as meeting initial inclusion criteria across the six cancer centers, we successfully located and spoke by phone with 1258 (74.7%); of these, 203 (16.1%) declined to participate. Of the 1055 survivors remaining, 592 (56.1%) reported a surveillance mammogram within the past 12 months, and thus, were not eligible, and 36 (3.4%) reported other radiographic breast cancer screening (MRI, PET, or CT scan) in the past 12 months and were also deemed ineligible. Thus, 628 (59.5%) of survivors willing to participate were not eligible, because they reported screening in the past 12 months. In addition, 314 (29.8%) were ineligible on the basis of substantial reported comorbidities, being older than age 75 years, or having had a bilateral mastectomy.

The remaining 113 breast cancer survivors (representing 10.7% of the 1055 agreeing to participate) met all inclusion criteria and were enrolled in the study. Of these, 95 (84.1%) successfully completed the baseline survey and were randomized to the high-intensity (50) or low-intensity (45) intervention. A follow-up assessment for receipt of mammography screening at three months was completed for 33 breast cancer survivors in the high-intensity arm and 25 in the low-intensity arm and at 12 months for 30 in the high-intensity arm and 24 in the low-intensity arm. Among the 58 survivors completing surveys at three months, eight (13.8%) did not complete a survey at 12 months, and among the 54 completing surveys at 12 months, four (7.4%) did not complete surveys at three months.

Calculation of screening adherence at three months and at 12 months for each intervention arm proceeded as follows. At three months, the adherence rate was simply the percent reporting screening mammography among those completing the three-month survey. For the adherence calculations at 12 months, a survivor was regarded as adherent if she had reported screening on either the three-month or 12-month survey (regardless of whether she completed one or both surveys). If she reported no screening at three months and did not take the 12-month survey, she was regarded as lost to follow-up.

First-Degree Relatives: Breast cancer survivors identified 394 FDRs with adequate contact information, and we successfully located and spoke with 313 (79.4%). Of these, 26 (8.3%) declined to participate. Of the 287 FDRs remaining, 132 (46.0%) reported having a screening mammogram within the past 12 months, and 48 (16.7%) were age 18–40 years and reported receiving a clinical breast exam within the past 12 months. Thus, 180 (62.7%) survivors willing to participate were not eligible, because they reported some form of breast screening in the past 12 months. In addition, 21 (7.3%) reported substantial comorbidities and were considered ineligible for the study.

The remaining 86 FDRs (representing 30.0% of the 287 agreeing to participate) were enrolled in the study. Of these, 83 (96.5%) completed the baseline survey and 35 were assigned to the high-intensity intervention arm and 48 to the low-intensity intervention arm. Follow-up was completed at three months for 24 FDRs in the high-intensity arm and 38 in the low-intensity arm and at 12 months for 23 in the high-intensity arm and 34 in the low-intensity arm. Among the 62 FDRs completing surveys at three months, nine (14.5%) did not complete a survey at 12 months, and among the 56 completing surveys at 12 months, three (5.4%) did not complete surveys at three months. Calculation of screening adherence at three months and 12 months for FDRs followed precisely the same decision rules as for survivors.

### 3.2. Intervention Impact

Breast Cancer Survivors: The randomized trial design was anticipated to achieve an adequate balance between arms in sociodemographic characteristics that might influence adherence to surveillance mammography. In Table 1, we find that adequate balance was achieved at baseline for all covariates except race/ethnicity (*p* = 0.02), wherein Black survivors were disproportionately represented in the high-intensity arm.

At study enrollment, almost 75% of survivors were ages 55 or younger, just over half were Black, just under half reported having a college or post-graduate degree, about 40% had annual family incomes greater than $50,000, and almost 60% were privately insured. About half were diagnosed with localized breast cancer, with the remainder of cases split between in situ and the regional stage.

**Figure 3:**
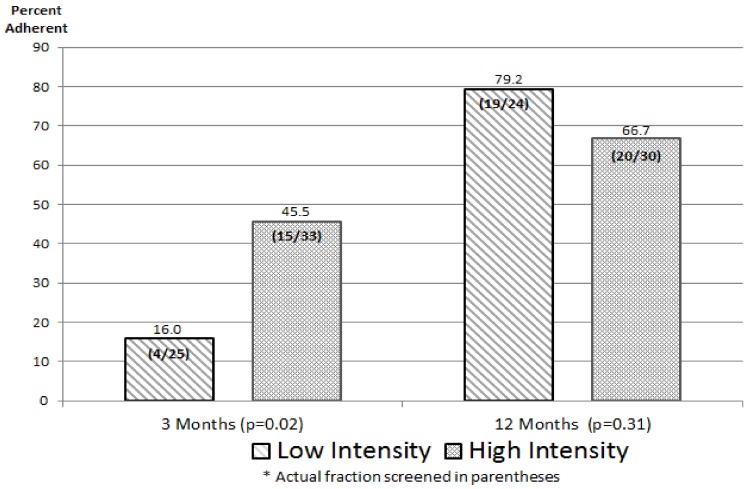
Intervention Impact on Mammography Screening Adherence Rates for Breast Cancer Survivors *.

The relative, and absolute, impacts of the interventions on achieving recommended breast cancer screening are summarized in Figure 3. At three months, survivors in the high-intensity arm were significantly more likely than survivors in the low-intensity arm to report having received a mammogram (45.5% vs. 16.0%, *p* = 0.02). However, at 12 months, there was no significant difference between the interventions (66.7% vs. 79.2%, *p* = 0.31). Thus, there was a rapid increase in screening in the high-intensity arm closely following the delivery of the multicomponent intervention. Between the three-month and 12-month reporting points, there were gains in both arms, but notably more so in the low-intensity arm. Pooling across arms, 39/54 (72.2%) of these survivors (all of whom were nonadherent at enrollment) had received surveillance mammography by month 12 of the study.

To investigate whether the sample attrition occurring between baseline and three months (see Figure 2) created new or substantial imbalances in covariate values between treatment arms, we replicated Table 1 for the 33 high-intensity and 25 low-intensity survivors participating in the three-month survey (table not displayed here). As at baseline, the only covariate with significant imbalance was race/ethnicity (0.05), with Blacks still disproportionately represented in the high-intensity arm. In a binary logistic regression model for the likelihood of screening adherence that included both intervention (high vs. low) and race/ethnicity (White vs. non-White), the latter variable was not close to significance (*p* = 0.52). This provides evidence that the modest degree of sample imbalance at three months did not significantly influence the apparent differential effect of interventions on screening outcomes for these breast cancer survivors.

Likewise, at 12 months, we replicated Table 1 to examine possible covariate imbalances for the 30 high-intensity and 24 low-intensity survivors (Figure 2). This time, we found no significant imbalances across covariates, lending support to the validity of the observed differential effect (or lack thereof) of interventions on screening outcomes for survivors at 12 months.

In addition, we examined whether there was a difference between intervention arms in having received a “reminder from your physician about getting a breast screening exam” (data not shown). At three months, about 53% of survivors in the high-intensity arm reported getting a reminder, compared with 42% in the low-intensity arm, but the difference was not significant (*p* = 0.42). At 12 months, about 62% of survivors in the high-intensity arm reported getting a reminder, compared with 48% in the low-intensity arm (*p* = 0.30).

**Figure 4:**
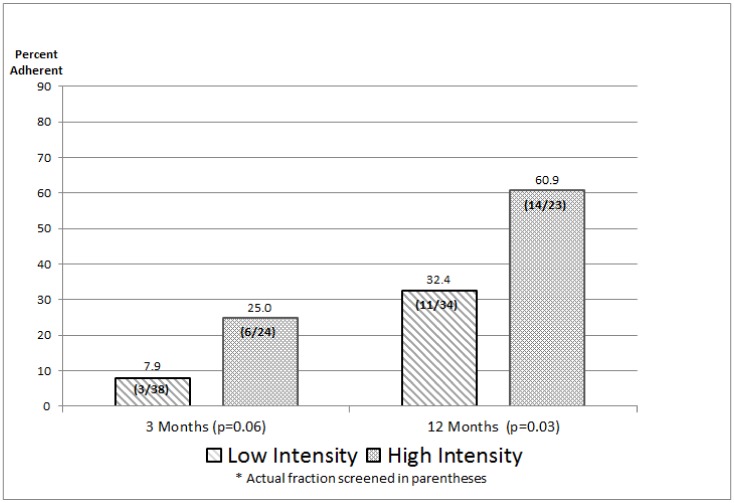
Intervention Impact on Mammography Screening Adherence Rates for FDRs.

First-Degree Relatives: There were no significant imbalances in covariate values between intervention arms for the FDRs at baseline (Table 2), nor were there imbalances at three months or 12 months (tables not shown here). As summarized in Figure 4, at three months, 25.0% of FDRs in the high-intensity arm reported getting a mammogram since study enrollment, compared with 7.9% in the low-intensity arm, although the difference was only approaching significance (*p* = 0.06). At 12 months, 60.9% in the high-intensity arm had received a mammogram, compared with 32.4% in the low-intensity arm (*p* = 0.03). Pooling across the arms, 25/57 (43.9%) of these FDRs (all nonadherent at enrollment) had received screening mammography by month 12.

Regarding receipt of a physician reminder about breast cancer screening (data not shown), at three months, about 42% of FDRs in the high-intensity arm and 32% of the low-intensity arm reported getting a reminder (*p* = 0.58). At 12 months, the corresponding figures were 59% and 39% (*p* = 0.15).

## 4. Discussion

Ever since publication of compelling evidence from clinical trials that screening mammography is associated with a reduction in breast cancer mortality, there has been much discussion about what constitutes appropriate risk-based screening. As Smith [23] and others have pointed out, a central challenge for population-level breast cancer screening is identifying those specific individuals at elevated risk and then encouraging appropriate, evidence-based screening.

### 4.1. Appraisal of Findings

In this study, we examined (a) whether data from population-based cancer registries can be deployed effectively in identifying, locating, and enrolling breast cancer survivors at elevated risks; (b) whether, with the assistance of these cancer survivors, we could identify and locate their female FDRs; and (c) whether brief (and potentially scalable) education-oriented interventions, delivered remotely by mail and phone, could successfully encourage recommended breast cancer screening among survivors and FDRs who were not adherent to these recommendations at the time of contact.

(a) Role of the State Cancer Registry: The Georgia Cancer Registry (and other high-quality population-based cancer registries) maintains very complete and accurate information on cancer characteristics, including tumor site, stage, sequence of the cancer, age at diagnosis, whether the woman with breast cancer has one or both breasts affected, and vital status. Consequently, we are highly confident that the study’s initial clinical inclusion criteria were successfully applied. Working closely with the six participating Georgia cancer centers and also using electronic search engines that can identify individuals’ current contact information, we were able to locate and attempt to make telephone contact with 74.7% (1258/1684) of breast cancer survivors meeting initial study inclusion criteria. Of these, 83.9% (1055/1258) agreed to participate, for an overall yield rate of 62.6% (1055/1684).

Several other studies have used state cancer registries to identify breast cancer survivors and to set the stage for interventions aimed at either the survivor, her FDRs, or both.

For a trial focused on increasing mammography screening among FDRs, Bastani et al. [8] randomly sampled 2500 breast cancer survivors diagnosed in 1988 from the California Cancer Registry, contacted them by mail only, and ended up with a sample of 1244 (49.7%) who met the requirement of having (potentially contactable) female relatives age 30 years or older.

To identify high-risk women for a planned future trial to increase use of cancer genetic services, Katapodi et al. [9] randomly sampled the Michigan Cancer Surveillance Program Registry for 3000 breast cancer survivors diagnosed at age 45 years or younger over the 1998–2012 period; ultimately, 33.2% of survivors who were potentially contactable (by mail) accepted participation in the study.

In a test of whether an academic medical center registry could be successfully used to identify survivors, Carpentier et al. [24] reported that 31.4% of breast cancer survivors responded to mail inquiries and 74% of these were willing to be contacted, implying a yield rate of 23.2%. In similar efforts for colon cancer, Glanz et al. [10] used the Hawaii Tumor Registry to identity 1612 survivors diagnosed from 1997–2001; they were able to contact 1098 and complete telephone interviews with 958, for an implied overall yield of 59.4%. Working with the Colorado Central Cancer Registry, Lowery et al. [25] received (mailed) survey responses back from 23% of all identified and located individuals diagnosed with colon cancer in Colorado during 2001–2005.

Through a combination of mail and intensive telephone recruitment, our study achieved a yield rate (about 63%) that was higher than many previous studies.

(b) Identifying First-Degree Relatives: As noted, because there is no registry or other available source of information to identify FDRs, the cancer survivor remains the critical link. In our study, of the 1055 breast cancer survivors interviewed, about a quarter reported having one or more study-eligible FDRs and supplied complete contact information for her—leading to an identification rate of 0.37 FDRs per survivor.

In Bastani et al. [8], the 1244 breast cancer survivors successfully contacted yielded a total of 1846 FDRs, or 1.48 FDRs per survivor. In Katapodi et al. [9], the 883 responding breast cancer survivors provided 442 identified FDRs, or 0.50 FDRs per survivor (though this reflected a study-imposed limit of two FDRs per survivor for the planned trial).

Clearly, an important question is how to engage most effectively with cancer survivors in identifying and making successful contact with their FDRs. In particular, could the FDR yield per survivor be increased significantly if the inquiry is made in a setting that is both more personal and professionally authoritative? A potentially suitable occasion for such conversations would be the survivorship care plan “hand-off” visit at the survivor’s cancer center. In that professional setting, the cancer treatment team could present information about the elevated risk status of FDRs and then strongly urge the survivor to encourage their FDRs to discuss cancer screening with their primary care providers.

(c) Intervention Impact on Breast Cancer Survivors: This is the first study, to our knowledge, reporting the results of a randomized trial comparing the effectiveness of educational interventions to improve breast cancer screening rates among breast cancer survivors who are both at elevated risk for additional breast cancer and currently nonadherent to screening recommendations.

At three months post-baseline, those in the high-intensity arm were significantly more likely to report receiving a mammogram since entering the study, compared with those in the low-intensity arm. However, at 12 months, there was no significant difference between arms. It is possible that the very act of personally intervening with these survivors―all of whom had experienced breast cancer and were now receiving phone calls and completing surveys about their screening behavior―was sufficient to trigger similar responses in both arms. In this regard, there is some evidence that the impact differential between more intensive, compared with less intensive, behavioral interventions may diminish over time [26].

For both arms together, about 72% of these survivors reported having had mammographic exams by 12 months. It is important to put this summary statistic in perspective. Since being nonadherent was a study inclusion criterion, it is possible that a portion of this very substantial jump in adherence is attributable to a “regression to the mean effect”. That is, some (unknown) number of these survivors would have ended up getting mammography in the 12-month window of their study enrollment even if they had received no intervention whatsoever, implying that the “true” intervention impact is less than the observable impact. As with many behavioral intervention trials, there was no “nonintervention” arm here, for both ethical and practical reasons. However, we noted also that the overall screening adherence rate among the 1055 survivors successfully interviewed for study eligibility was 59.5%. If one regards this as a reasonable estimate of the nonintervention screening rate among such breast cancer survivors in metropolitan Atlanta and Southwest Georgia during the study period, we can conclude that our interventions spurred screening among nonadherent survivors to rates that somewhat exceeded the natural “nonintervention” rate. Finally, even if a regression effect was in play, there is little reason to believe it would have affected the high and low-intensity arms differently in this randomized trial; hence, differences in intervention impact should not have been influenced appreciably.

One additional, though limited, perspective on these screening rates for our Georgia-based survivors can be found in national data. The estimated rate of mammography screening adherence (“every 1–2 years” following recommendations from the U.S. Preventive Services Task Force) among breast cancer survivors in the U.S. based on self-reported data from the National Health Interview Survey ranged from about 74% to 78% during 2000 to 2010 [27].

(d) Intervention Impact on First-Degree Relatives: We found that a higher percentage of FDRs in the high-intensity (compared with low-intensity) arm received mammography, both at three months and at 12 months. Since these women were not screening adherent at baseline and there is again the possibility of a regression effect, it is useful to put the rate increases into perspective. The breast cancer screening rate among all FDRs successfully interviewed for study eligibility was 61.3%. If we regard this as an estimate of the screening rate for a hypothetical nonintervention arm, then the high-intensity intervention led to a screening rate among (these nonadherent) FDRs at 12 months (60.9%) that is quite comparable to the rate for all successfully surveyed FDRs. By comparison, data from the CDC’s Behavioral Risk Factor Surveillance System (BRFSS) for 2014 indicated that 59.8% of surveyed women in Georgia age 40 or older reported having a mammogram within the past 12 months [28]. We would expect the screening rate for our FDRs to be at least this large, since all are in families touched by breast cancer.

Yet another perspective is provided by the comprehensive meta-analyses published in 1999 by Yabroff and Mandelblatt [29] and Mandelblatt and Yabroff [30] on the impact of various approaches to enhancing breast cancer screening among average-risk women. While findings varied widely, the influence of behavioral interventions on mammography screening compared with usual care tended to center at about 13 percentage points. By comparison, at 12 months in our study, the percentage difference in screening between FDRs in the high-intensity and low-intensity interventions was 28.5 (60.9–32.4%) percentage points.

In terms of appraising the impact of interventions to improve mammography rates among FDRs of breast cancer survivors, the best published study for comparison appears to be Bastani et al. [8], although it is set in a different time period (the early 1990s) and place (California). In that study, the 902 FDRs identified were randomized to an intervention group receiving a mail-out package that included a personalized risk assessment, other materials tailored for high-risk women, and a message about the importance of a regular mammography; a control group received NCI-produced materials on breast cancer and screening mammography. Unlike our study, being adherent to screening recommendations at baseline was not an exclusion criterion. The impact of the intervention at 12 months post-baseline, with 753 FDRs still participating, was to increase screening rates 10.2 percentage points, from 55.0% to 65.2%; there was a 2.5 percentage point improvement in the control arm, from 54.9% to 57.7%, and the overall difference between arms was significant (*p* = 0.05). In our study, there was a much greater difference between the high-intensity and low-intensity arms at the 12-month follow-up, although the mammography screening rate for high-intensity (60.9%) was in the range of that reported by Bastani et al. for their intervention arm (65.2%). Overall, both studies found that a focused intervention significantly increased FDR screening rates.

### 4.2. Study Limitations

Accuracy of Screening Reports: Our study relied on self-reports about the occurrence and the timing of mammography, similar to many other studies [8,10] and surveys [27,28]. The accuracy of these reports for the general at-risk population of women has been appraised in meta-analyses conducted by Howard et al. [31] and Rauscher et al. [32]; by Cronin et al. [33] in a comparison of self-reports of mammography screening in Vermont with rates computed from a high-quality screening registry covering that state and also screening rates modeled on data from the National Health Interview Survey; and, more recently, by Tiro et al. [34] in a single-institution study that compared mammography rates for breast cancer survivors based on their self-reports with data from electronic health records. Across studies, a recurrent finding is that screening rates based on patient self-reports tend to exceed rates for the same or comparable individuals derived from medical records.

For breast cancer survivors, Tiro et al. found very high sensitivity (99%)—meaning that mammography actually received within a specific timeframe was reported correctly—but low specificity (31%), implying a significant tendency to report mammography when it had not been received. These data lead to a positive predictive value (PPV) of 89% (if the survivor reported mammography, the probability she received it is 0.89) and a negative predicted value (NPV) of 82% (if the survivor said she did not get mammography, there is a 0.82 probability that was correct). Applied to our study, this PPV suggests that about 11% of survivors excluded from enrollment because they reported having a mammogram (in the past 12 months) should have been included. At the same time, this NPV indicates that about 18% of survivors included in the study because they reported not having a mammogram should not have been included. That said, whatever the precise degree of reporting error in our study, the randomization design implies we should expect any such errors to affect the high and low-intensity arms similarly.

Findings from Howard et al. [31], Rauscher et al. [32], and Cronin et al. [33] regarding the accuracy of mammography reports from at-risk women in general arguably can be applied to the FDRs in our study. Again, to the degree mammography was overreported by FDRs, there is no reason to suspect an imbalance across the high and low-intensity arms.

Across these studies, it is invariably assumed that clinical documentation of screening (or its absence) is the gold standard, though there is a non-zero chance, especially in busy health systems, that a mammogram is not properly charted. Looking to the future, to the extent that reports of screening by survivors and FDRs can be verified through (IRB-approved) access to their medical records and health insurance claims data, concerns about accuracy could be significantly reduced.

Other Data and Statistical Considerations: There are several points to note. First, most—but not all—survivors and FDRs randomized to the high-intensity arm were able and willing to identify a primary care provider, who would then be sent the information packet encouraging screening. Specifically, we had sufficient information to conduct PCP outreach for 47 of the 50 survivors enrolled at baseline (94.0%) and for 27 of the 35 FDRs enrolled at baseline (77.1%). This suggests that the observed difference between high-intensity and low-intensity is possibly conservative, compared to the ideal in which PCP outreach had been completed for everyone in the high-intensity arm.

Second, it would be interesting in principle to examine whether there was a significant correlation between the screening behavior of a survivor and her FDR(s), especially since the study was designed to ensure that an FDR would be assigned to the same intervention to which her survivor-relative had been randomized. In reality, however, there were only seven survivor-FDR dyads available among the 95 survivors and 83 FDRs who met all study inclusion criteria and were enrolled at baseline—far too few for a meaningful analysis. In retrospect, this lack of overlap should not be as surprising as it first may seem, since only a fraction of survivors identified FDRs (see discussion just above), and many survivors naming FDRs were not among the 95 survivors finally enrolled at baseline, given the study’s inclusion/exclusion criteria.

Third, there is the difficult question that naturally arises in any such intervention trial regarding whether the sample sizes that finally emerged—for survivors and FDRs at three months and at 12 months—were sufficient for drawing clear, convincing conclusions about intervention impact. In trials, the standard approach is to ensure that the study is “adequately powered” to identify “clinically meaningful” intervention differences when they exist. In the present study, establishing expectations about needed and expected sample sizes was particularly challenging, given the range of ex ante unknowns. Beyond the report from the Georgia Cancer Registry on the number of survivors available over the study period, we had little information to gauge how many of these survivors could be located, would consent to be interviewed, meet study inclusion criteria, complete baseline documentation and receipt of intervention, and then be available for interviews at three months and 12 months (Figure 2). Moreover, both the number of FDRs per survivor and the willingness of survivors to identify FDRs were unclear. In response, our pre-study power calculations were highly conditional and conducted more in the form of sensitivity analyses (e.g.; if x and y hold, then z cases were needed where there were multiple x’s and y’s).

In the end, the sample sizes that emerged were sufficiently large to draw the bottom-line inferences summarized in Figure 3 and Figure 4. In three of the four comparisons, there was a quantitatively notable and statistically strong difference between the high-intensity and low-intensity interventions, and in the anticipated direction. For survivors at 12 months, the difference between interventions was not significant (*p* = 0.31), nor in the anticipated direction. It can be shown that for this difference (66.7% high-intensity vs. 79.2% for low-intensity) to be statistically significant at *p* = 0.05, it would require about 185 patients per arm—far beyond what we achieved or, in fact, anticipated. On balance, we believe it is reasonable to conclude that the three statistically strong intervention differences in Figure 3 and Figure 4 are large enough to be also “clinically important”, while the results for survivors at 12 months does not pass that (admittedly judgmental) test.

Fourth, the extent of participant drop-out—especially between the three-month and 12-month surveys (Figure 2)—is a concern that should be addressed aggressively in future studies that extend this line of analysis. Even if one can demonstrate that such drop-out does not lead to significant covariate imbalances between intervention arms (as we did), there is still the risk that arms will come to differ in terms of unmeasured confounders, notwithstanding a successful initial randomization. In our view, the best preventative action going forward would be a study design variant that calls for strong, ongoing engagement by health care providers—the study participants’ oncologists and PCPs—to offer encouragement to stay the course and (of course) be screened. Such proactive engagement could take the form of periodic mail/e-mail communication, amplified during clinic visits.

As a final matter, it is interesting to note the 12-month screening rates achieved with the low-intensity intervention (the CDC pamphlet encouraging screening and breast health): 79.2% among survivors and 32.4% among FDRs. Given that this intervention strategy is clearly “low-cost” compared to the multipronged high-intensity intervention, future studies could evaluate the cost-effectiveness of high vs. low, both in terms of screening adherence and (in more complex analyses) of impact on breast cancer outcomes.

## 5. Implications for Improving Adherence to Breast Cancer Screening

### Recommendations

Breast Cancer Survivors: As noted, the American College of Surgeons’ Commission on Cancer and other major cancer professional organizations now regard receipt of a survivorship care plan by patients completing initial therapy as a central component of quality of care [15,16,17,18,19]. Moreover, in the United States, roughly 70% of all initial therapy for major cancer sites, including breast cancer, is provided in CoC facilities. Hence, for the majority of breast cancer patients completing therapy, the most efficient, if not also most effective, approach for delivering the message about recommended surveillance mammography is to make it an integral part of the survivorship care plan, especially during the “hand-off” meeting with oncology providers. In addition, the oncology team could ensure that the survivor’s primary care provider(s) receive a copy of the survivorship care plan, noting in particular whether the survivor (or her close relatives) is at elevated risk for breast cancer because of an early-age diagnosis or bilateral disease. There is already a clear emphasis on providing primary care provider(s) with a copy of the survivor’s survivorship care plan [35,36].

However, for the cancer survivor who did not receive a survivorship care plan upon completion of initial cancer therapy, an outreach-oriented intervention strategy of the type examined here represents a viable approach to encourage guideline breast cancer screening. Identifying the survivor through a population-based cancer registry is central to this strategy, and such registries are not only in operation across all of North America but in virtually all European countries, in Latin America, across Asia, in Australia and New Zealand, and in many African countries, according to the International Association of Cancer Registries (IACR) [37]. Hence, our strategy for identifying high-risk cancer survivors to receive education-oriented interventions to improve surveillance screening and also other health-enhancing behaviors could (with appropriate tailoring) be embraced in countries across the globe.

First-Degree Relatives: Since the only practical pathway to the FDR is through the cancer survivor, how might this engagement best be accomplished? As noted, one strategy is to make counseling about the cancer risks of close relatives an integral component of the survivorship care plan “hand-off” meeting. Such information could be used subsequently as a springboard for inviting the survivor to work with health care providers and others (including public agencies, if applicable) to make contact with her FDRs. This could take the form of the survivor supplying the contact information directly, as in our study and in Bastani et al.; or the survivor making the commitment to reach out to the FDR directly to encourage breast cancer screening, as was the strategy in Katapodi et al. While the major survivorship care plan templates have not emphasized this type of family outreach effort, it merits pilot testing, especially as the percentage of survivors receiving survivorship care plans continues to grow.

## 6. Conclusions

We conducted a randomized trial that demonstrated the feasibility and potential effectiveness of using the state cancer registry as a platform for interventions to increase recommended breast cancer screening among breast cancer survivors and their close relatives. For survivors and FDRs who were not already screening adherent at study enrollment, the interventions led to substantial overall increases in mammography screening rates, but the influence of the “intensity” of the intervention differed between groups. For survivors, there was no difference between high-intensity and low-intensity at 12 months following randomization. For FDRs, those receiving the high-intensity intervention were significantly more likely than those receiving the low-intensity intervention to be screening adherent at 12 months. Looking ahead, the focus should be on how to deliver the message “get screened” most effectively and efficiently, capitalizing on the population-based cancer registry as a platform for engaging the survivor, who then provides the gateway to family members at elevated risks of cancer.

## Figures and Tables

**Table 1:** Sociodemographic characteristics among high-risk breast cancer survivors by randomized screening intervention groups.

Characteristics	Level		Intervention Group		
		Sample Totals ^a^	Low-Intensity ^a^ *N* = 45	High-Intensity ^a^ *N* = 50	*p*-Value *
Age	≤45 years	19 (20.0)	10 (22.2)	9 (18.0)	0.673
	46−55 years	51 (53.7)	22 (48.9)	29 (58.0)	
	≥56 years	25 (26.7)	13 (28.9)	12 (24.0)	
Race/ethnicity	White	33 (35.1)	16 (35.6)	17 (34.7)	0.022
	Black	49 (52.1)	19 (42.2)	30 (61.2)	
	Other	12 (12.8)	10 (22.2)	2 (4.1)	
Marital status	Married/partnership	49 (52.1)	22 (48.9)	27 (55.1)	0.547
	Other	45 (47.9)	23 (51.1)	22 (44.9)	
Education level (highest grade completed)	College or post-grad	45 (48.4)	23 (52.3)	22 (44.9)	0.477
	Other	48 (51.6)	21 (47.7)	27 (55.1)	
Employment status	Working (FT/PT)	55 (59.1)	29 (65.9)	26 (53.1)	0.208
	Other	38 (40.9)	15 (34.1)	23 (46.9)	
Annual family income	<$50,000	43 (45.7)	18 (40.0)	25 (51.0)	0.110
	≥$50,000	36 (38.3)	22 (48.9)	14 (28.6)	
	Refused/DK	15 (16.0)	5 (11.1)	10 (20.4)	
Health insurance status ^b^	Private	54 (59.3)	28 (65.1)	26 (54.2)	0.537
	Public	16 (17.6)	7 (16.3)	9 (18.8)	
	Uninsured	21 (23.1)	8 (18.6)	13 (27.0)	
SEER Summary Stage ^c^	In Situ	22 (23.1)	11 (24.4)	11 (22.0)	0.874
	Localized	47 (49.5)	21 (46.7)	26 (52.0)	
	Regional	26 (27.4)	13 (28.9)	13 (26.0)	
Year of Diagnosis	2000−2002	28 (29.5)	11 (24.4)	17 (34.0)	0.486
	2003−2006	38 (40.0)	18 (40.0)	20 (40.0)	
	2007−2009	29 (30.5)	16 (35.6)	13 (26.0)	

* Calculated for two-sided chi-square test of the null of no association between the characteristic and randomization group; *p* < 0.05 is regarded as indicating a significant association (and bolded). ^a^ For a given characteristic, the number of participants may not sum to the total, due to nonresponse. ^b^ Private insurance also includes Medicare with private supplemental (“Medigap”) insurance. Public insurance includes Medicare only, Medicaid, Veterans Affairs (VA), or TRICARE (military) coverage. SEER = Surveillance, Epidemiology, and End-Results. Numbers in parentheses are percentages. ^c^ Women diagnosed with distant or unknown stage breast cancers were excluded from the study.

**Table 2:** Sociodemographic characteristics among first-degree relatives (FDRs) of high-risk breast cancer survivors by randomized screening interventions.

			Intervention Group		
Characteristics	Level	Sample Totals ^a^	Low-Intensity ^a^ *N* = 48	High-Intensity ^a^ *N* = 35	*p*-Value *
Age	≤45 years	52 (62.7)	31 (64.6)	21 (60.0)	0.646
	46−55 years	13 (15.6)	6 (12.5)	7 (20.0)	
	≥56 years	18 (21.7)	11 (22.9)	7 (20.0)	
Race/ethnicity	White	39 (47.5)	22 (45.8)	17 (50.0)	0.330
	Black	40 (48.8)	23 (47.9)	17 (50.0)	
	Other	3 (3.7)	3 (6.3)	0 (0.0)	
Marital status	Married/partnership	32 (39.5)	20 (41.7)	12 (36.4)	0.631
	Other	49 (60.5)	28 (58.3)	21 (63.6)	
Education level (highest grade completed)	College or post-grad	41 (50.0)	23 (47.9)	18 (47.1)	0.939
	Other	41 (50.0)	25 (52.1)	16 (52.9)	
Employment status	Working (FT/PT)	53 (66.3)	29 (61.7)	24 (72.7)	0.305
	Other	27 (33.7)	18 (38.3)	9 (27.3)	
Annual family income	<$50,000	36 (43.9)	21 (43.8)	15 (44.1)	0.289
	≥$50,000	39 (47.6)	21 (43.8)	18 (53.0)	
	Refused/DK	7 (8.5)	6 (12.4)	1 (2.9)	
Health insurance status ^b^	Private	47 (57.3)	27 (57.4)	20 (57.1)	0.614
	Public	15 (18.3)	10 (21.3)	5 (14.3)	
	Uninsured	20 (24.4)	10 (21.3)	10 (28.6)	

* Calculated for two-sided chi-square test of the null of no association between the characteristic and randomization group; *p* < 0.05 is regarded as indicating a significant association. Numbers in parentheses are percentages. ^a^ For a given characteristic, the number of participants may not sum to the total, due to nonresponse. ^b^ Private insurance also includes Medicare with private supplemental (“Medigap”) insurance. Public insurance includes Medicare only, Medicaid, Veterans Affairs (VA), or TRICARE (military) coverage.
